# Foam nickel-PDMS composite film based triboelectric nanogenerator for speed and acceleration sensing

**DOI:** 10.1016/j.heliyon.2023.e17467

**Published:** 2023-06-21

**Authors:** Wang Peng, Qianqiu Ni, Linfeng He, Qingxi Liao

**Affiliations:** a College of Engineering, Huazhong Agricultural University, Wuhan, 430070, China; b Shenzhen Branch, Guangdong Laboratory for Lingnan Modern Agriculture, Genome Analysis Laboratory of the Ministry of Agriculture, Agricultural Genomics Institute at Shenzhen, Chinese Academy of Agricultural Sciences, Shenzhen, 518000, China; c Shenzhen Institute of Nutrition and Health, Huazhong Agricultural University, Wuhan, 430070, China; d Key Laboratory of Agricultural Equipment in Mid-Lower Yangtze River, Ministry of Agriculture and Rural Affairs, Wuhan, 430070, China; e The Center of Crop Nanobiotechnology, Huazhong Agricultural University, Wuhan, 430070, China; f School of Mechanical Science and Engineering, Huazhong University of Science and Technology, Wuhan, 430074, China

**Keywords:** Triboelectric nanogenerator, PDMS, Self-powered sensing, Speed, Acceleration

## Abstract

As a new energy conversion technology, triboelectric nanogenerator (TENG) can use the coupling of triboelectrification and electrostatic induction effect to convert tiny mechanical energy into electrical energy, powering small electronic devices. In this paper, a vibration sensing triboelectric nanogenerator (V-TENG) based on a foam nickel-PDMS composite film was prepared, which can convert low frequency and small-amplitude mechanical energy into electrical energy, and the open circuit voltage of V-TENG can reach 3.6V at a vibration frequency of 4 Hz. In addition, the V-TENG can be used as a self-powered speed/acceleration sensor to detect speed changes in the range of 0.3 m/s to 1.5 m/s and acceleration changes in the range of 3 m/s^2^ to 13 m/s^2^.

## 1 Introduction

With the increasing demand for energy and threaten of environmental pollution, the development and utilization of green and sustainable energy has attracted great attention, among which the use of distributed, wireless and disorderly energy in the environment to power sensors has become a hot research topic. As a new energy conversion technology [[1], [2], [3]], the emergence of triboelectric nanogenerator (TENG) has brought new research directions for the collection of low-frequency energy such as micro-mechanical energy, wind energy, and wave energy [[4], [5], [6]]. Triboelectric nanogenerator have the advantages of high output, wide range of applications, low cost, light weight, flexible structural design, etc., and have great potential in the design and manufacture of energy collection and self-powered sensors [[7], [8], [9]]. At present, there have been many studies of self-powered sensors based on triboelectric nanogenerators. For example, the self-powered sensors that collect wind energy are mostly wind models or flag types, which can be used to detect wind speed and wind direction [[10], [11], [12], [13], [14], [15]]. Self-powered sensor for detecting stress strain, which can detect stress strain such as tensile force and pressure [[16], [17], [18], [19], [20], [21]]. Self-powered sensor for detecting chemicals [22,23]. Sensors that collect sound energy to achieve self-powered sound sensing [24,25].

In addition, triboelectric nanogenerators can also be applied to self-powered motion detection, Yi et al. reported a self-powered single-electrode triboelectric sensor for monitoring two-dimensional motion, which can detect the trajectory, velocity and acceleration of moving objects in real time [26]. Guo et al. proposed a self-powered three-dimensional motion sensor with a unique mesh cross-electrode structure design that can obtain three-dimensional motion information about an object, including pressure, velocity, and trajectory [27]. Si et al. developed a new, easy-to-process self-driven triboelectric multi-information motion monitoring sensor based on a single-electrode working mode that can accurately determine the velocity and acceleration of an object as it moves on the sensor surface [28]. Heo et al. proposed a practical reducer triboelectric nanogenerator that can convert the dynamic motion of the wheel through the speed reduction belt into an electrical signal to achieve self-powered speed sensing and alarm functions [29]. Chen et al. proposed a self-powered flexible triboelectric sensor for finger trajectory sensing that tracks continuous sliding information on the fingertip, such as trajectory, velocity, and acceleration [30]. However, these triboelectric sensors generally have the problems of cumbersome manufacturing steps and complex structure. Therefore, it is necessary to design and manufacture a triboelectric motion sensor with simple structure and fabrication.

In this paper, a vibration-sensing triboelectric nanogenerator (V-TENG) based on a foam nickel-PDMS composite film and a nylon ball was fabricated. When V-TENG vibrates, the nylon ball will be separated from the foam nickel-PDMS composite film in contact, due to the triboelectrification effect and electrostatic induction effect, the nylon ball will produce the same amount of opposite charge with the foam nickel-PDMS composite film surface, as the nylon ball moves, the potential of the foam nickel electrode will change, by changing the frequency, speed or acceleration of the vibration, different output signals can be obtained. As a result, the V-TENG can be used as a self-powered speed/acceleration sensor with potential applications in motion monitoring.

## 2 Results and discussion

### 2.1 Structure and working principle

**Fig. 1:**
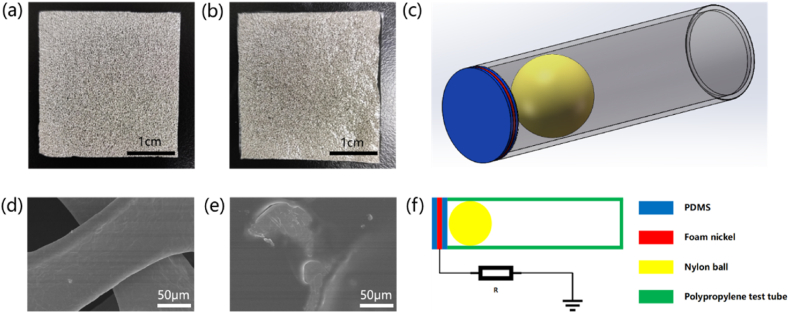
Materials and structures of V-TENG. (a) Foam nickel, (b)foam nickel-PDMS composite film, (c) V-TENG model diagram, (d) SEM image of foam nickel, (e) SEM image of foam nickel-PDMS composite film, (f) V-TENG structural diagram.

As a triboelectrically negative material with strong electron acquisition ability, PDMS is widely used in many triboelectric nanogenerators. In this study, a foam nickel-PDMS composite film was prepared, by applying PDMS to the foam nickel, using the rough porous structure of the foam nickel, to obtain a PDMS film with a large number of surface microstructures, increase the contact area of the PDMS film, and increase the surface charge density of the PDMS film after contact. At the same time, foam nickel has good electrical conductivity and can be used as an electrode material for PDMS films. Fig. 1a and b are pictures of nickel foam and foam nickel-PDMS composite films, respectively. In addition, Fig. 1d and e are scanning electron microscope images of foam nickel and foam nickel-PDMS composite films, respectively. It can be seen that the surface microstructure of foam nickel is transferred to the surface of PDMS, which increases the surface contact area of PDMS.

**Fig. 2:**
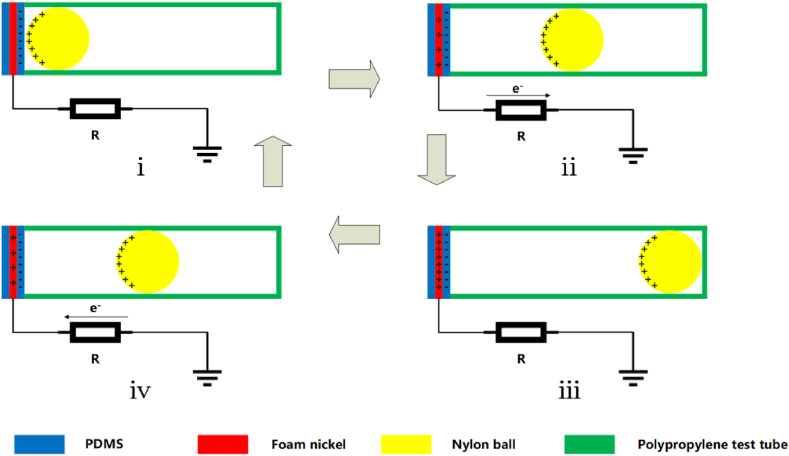
Schematic illustration of working principles of V-TENG, (i–iv) Charge distribution of the V-TENG during a complete cycle i) contact state ii) separate state iii) the farthest distance in separation state and iv) approaching state.

The structure of V-TENG is shown in Fig. 1c and f, and the triboelectric nanogenerator consists of a foam nickel-PDMS composite film, a nylon ball and a polypropylene test tube. Polypropylene test tubes are used as housings, foam nickel-PDMS composite film is used as triboelectrically negative material, and nylon is used as triboelectrically positive material. When the triboelectric nanogenerator vibrates in a horizontal direction, the nylon ball separates from the foam nickel-PDMS film in contact, and then the transfer of charge and flow of electrons occur. The specific working principle is shown in Fig. 2(i-iv). When the triboelectric nanogenerator is in the initial state (i), the nylon ball is in contact with the PDMS film. Due to the triboelectrification and electrostatic induction effect, the nylon ball and the foam nickel-PDMS composite film carry an equal amount of opposite charge, and they are in the electrostatic equilibrium state. When the nylon ball is far away from the PDMS film (i-ⅲ), the potential between the two gradually increases. In order to balance the potential difference, the nickel foam electrode induce the corresponding positive charge based on electrostatic induction. When electrons flow out of the foam nickel electrode, the current forms in external circuit. When the nylon ball moves to the farthest distance from the PDMS film (ⅲ), the potential difference between the two is the largest. In this situation, the foam nickel and PDMS film is still in the electrostatic equilibrium state with an equal amount of opposite charge. When the nylon ball re-approach the PDMS film (ⅲ-ⅰ), the potential difference between the two gradually decreases. The positive charge on the nylon balls gradually reaches an electrostatic equilibrium with the negative charge on the PDMS film. The electrons flow into the foam nickel electrode, and forms the opposite current in external circuit. When the nylon ball moves to the closest point to the PDMS film, the potential difference between the two is minimal (ⅰ). When the nylon ball repeats this periodic motion, it produces a continuous alternating current output.

**Fig. 3:**
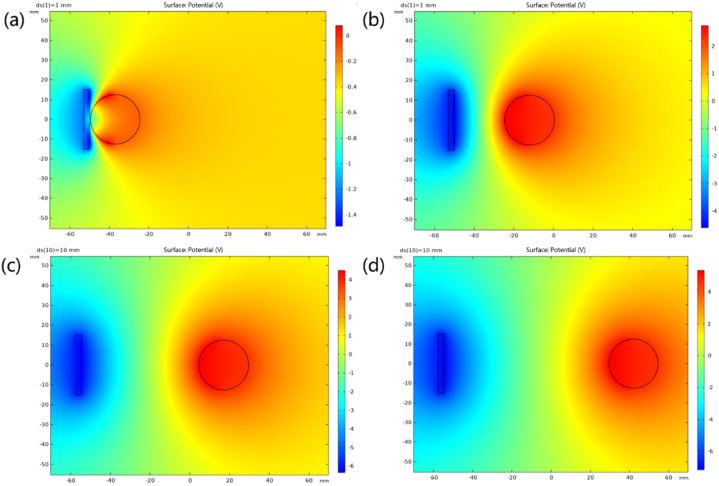
Simulation of potential distribution of different states of V-TENG. (a) 0 mm (b)25 mm (c)50 mm (d)75 mm.

The electrostatics module of COMSOL software was used to simulate the triboelectric nanogenerator working principle. The pitch between the nylon ball and the PDMS is set as 0 mm, 25 mm, 50 mm, and 75 mm, respectively, as shown in Fig. 3a, b, Fig. 3c and d respectively. It can be seen that as the distance increases, the unilateral voltage increases from about 0V to 4V, which is in line with the inference of the potential change during V-TENG motion.

### 2.2 Output performance

**Fig. 4:**
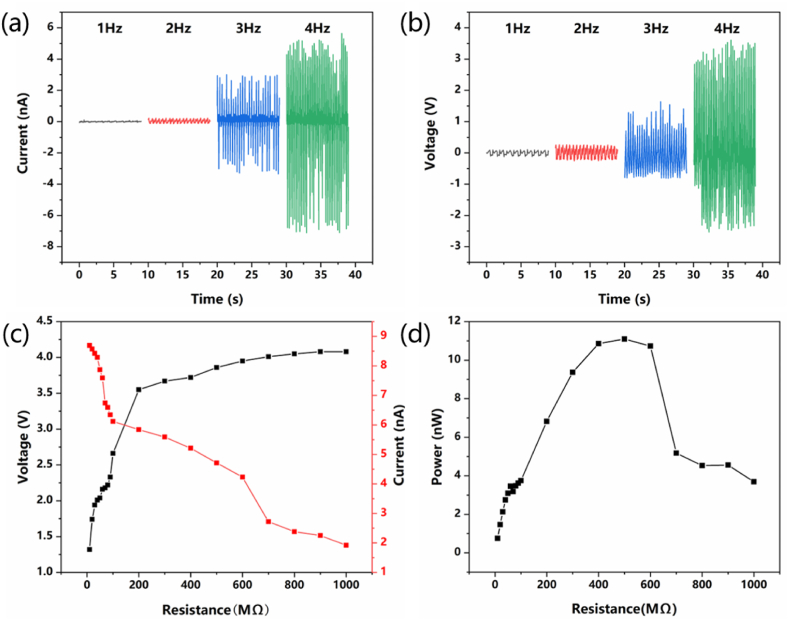
Output performance of V-TENG at different vibration frequencies and load resistances. (a) Frequency and short-circuit current (b) Frequency and open-circuit voltage (c) Load resistance and open-circuit voltage, Short-circuit current (d) Load resistance and power.

In order to test the output performance of the V-TENG, it is fixed to the linear motor. The linear motor is controlled to vibrate at different frequencies, and the open circuit-voltage and short-circuit current of the V-TENG are measured using a multimeter. When the vibration frequency increases, the open-circuit voltage and short-circuit current of V-TENG are gradually increasing. When the vibration frequency is 4 Hz, the peak open-circuit voltage can reach 3.6V, and the peak short-circuit current can reach 7.1 nA, as shown in Fig. 4a and b. In order to study the electrical performance of V-TENG under different load resistances, keep the vibration frequency of V-TENG constant at 4 Hz and change the external load resistance. As can be seen from Fig. 4c and d, when the external load resistance gradually increases from 0Ω to 1000 MΩ, the open-circuit voltage of V-TENG gradually rises from 1.3V to 4.1V, the short-circuit current gradually drops from 8.7 nA to 1.9 nA. When the external load resistance is 500 MΩ, the instantaneous output power is maximized with 11.1 nW, means the best matched external resistance is about 500 MΩ. The instantaneous output power is calculated by Eq. (1):

(1) $$\begin{array}{c} P=I_{t}^{2}R\# \end{array}$$

where R is the loaded resistance and I_t_ is the instantaneous current across the resistance.

### 2.3 Speed/acceleration detection

The foam nickel-PDMS composite film has a certain flexibility. When the nylon ball hits the foam nickel-PDMS composite film with different forces, the degree of deformation of the composite film is different. The contact area will change, and the amount of transferred charge will also change. By controlling the change in the impact force of the nylon ball and the composite film, different voltage and current outputs can be obtained. The vibration speed or acceleration of V-TENG determines the impact force when the nylon ball contacts with the foam nickel-PDMS composite film. Therefore, the V-TENG can be used as a self-powered sensor to detect vibration speed or acceleration.

**Fig. 5:**
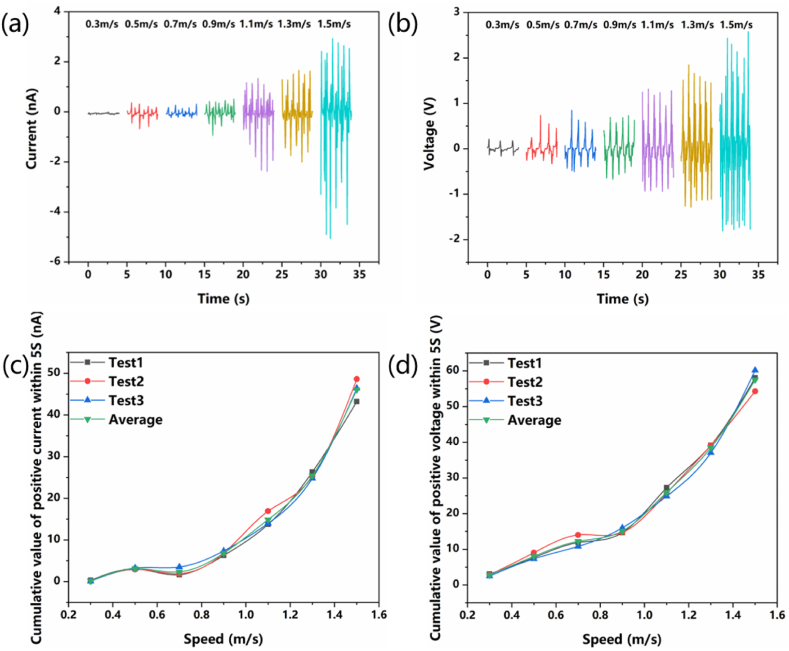
The relationship between the open-circuit voltage and short-circuit current and speed of V-TENG. (a) Short-circuit current (b) Open-circuit voltage (c) Positive current accumulation value within 5s (d) Positive voltage accumulation value within 5s.

In order to study the relationship between the vibration speed of V-TENG and its output voltage and current, fix V-TENG on the linear motor, gradually increase the vibration speed of the linear motor the vibration speed of the linear motor, and measure the output of V-TENG. As shown in Fig. 5a and b, when the vibration speed reaches 0.3 m/s, the nylon ball in V-TENG reaches a stable motion state and can periodically contact and separate from the foam nickel-PDMS composite film. At this time, the short-circuit current and open-circuit voltage of V-TENG are 0.1 nA and 0.3V respectively. With the increase of vibration speed, the short-circuit current and open-circuit voltage increase continuously, reaching the maximum values of 5.6 nA and 2.6V respectively at 1.5 m/s. When the vibration speed exceeds 1.5 m/s, the movement of the nylon ball presents an irregular state.

After V-TENG vibrates for 5 s, the positive values in the measured current data are accumulated, and the relationship between the accumulated value and the vibration speed is shown in Fig. 5c. From the results of the three tests and their average values, it can be seen that with the increase of vibration speed, the accumulated value of current also increases, and the two have a certain linear relationship. Similar to the accumulated value of current, the accumulated value of voltage and vibration speed also have such a linear relationship, as shown in Fig. 5d. Therefore, the vibration speed of the TENG can be determined by detecting the short-circuit current or the open-circuit voltage of the V-TENG.

**Fig. 6:**
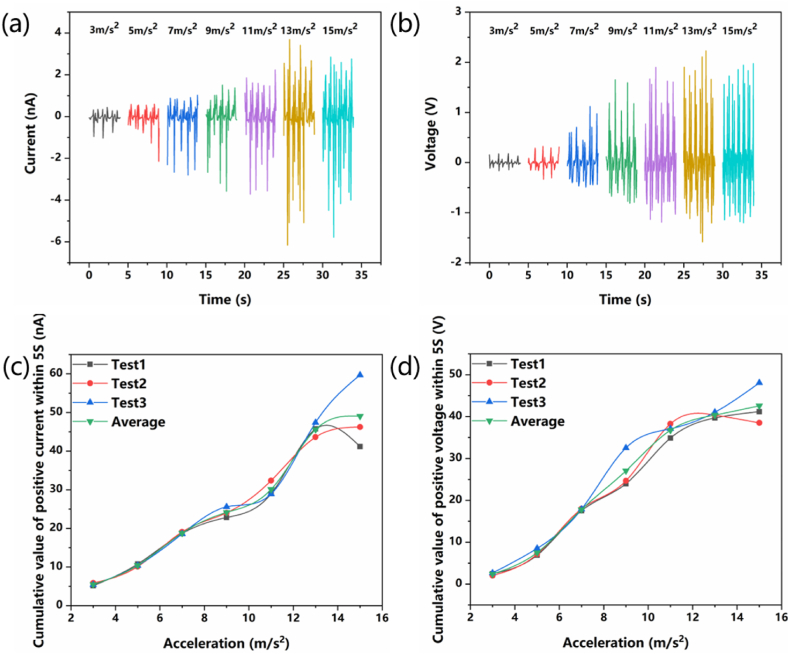
The relationship between the open-circuit voltages, short-circuit current and acceleration of V-TENG. (a) Short-circuit current (b) Open circuit voltage (c) Positive current accumulation value within 5s (d) Positive voltage accumulation value within 5s.

The same method as above has been used to study the relationship between the vibration acceleration of V-TENG and its output voltage and current. As shown in Fig. 6a and b, When the vibration acceleration gradually increases from 3 m/s^2^ to 13 m/s^2^, the short-circuit current gradually increases from 0.4 nA to the maximum value of 6.2 nA, and the open-circuit voltage gradually increases from 0.3V to the maximum value of 2.3V. When the vibration acceleration continues to increase to 15 m/s^2^, the short-circuit current and open-circuit voltage drop to 5.8 nA and 2.0V respectively, which may be due to insufficient contact between nylon ball and foam nickel-PDMS composite film under excessive acceleration. When the vibration speed exceeds 15 m/s^2^, the movement of the nylon ball presents an irregular state. After V-TENG vibrates for 5 s, the positive values in the measured current and voltage data are accumulated respectively. The relationship between them and the vibration acceleration is shown in Fig. 6c and d. In the range of 3 m/s^2^ to 13 m/s^2^, with the increase of the vibration acceleration, the cumulative values of current and voltage increase, which has a certain linear relationship. Therefore, the vibration acceleration of the TENG can be determined by detecting the short-circuit current or the open-circuit voltage of the V-TENG.

Through the above experiments, it can be determined that there is a certain linear relationship between the output voltage and current of V-TENG and the speed/acceleration. V-TENG can be horizontally installed on moving objects to detect the speed change from 0.3 m/s to 1.5 m/s and the acceleration change from 3 m/s^2^ to 13 m/s^2^.

## 3 Conclusion

In this paper, a vibration sensing triboelectric nanogenerator (V-TENG) was prepared by using a foam nickel-PDMS composite film as a triboelectric layer. V-TENG has a simple structure and can collect micro-mechanical energy. The output voltage and short-circuit current of V-TENG reached 3.6V and 7.1 nA at a vibration frequency of 4 Hz. When the external load resistance is 500 MΩ, the instantaneous peak power density is 11.1 nW. V-TENG can be used as a self-powered speed/acceleration sensor to detect vibration speed and vibration acceleration, and the sensing range of vibration speed and acceleration is 0.3 m/s to 1.5 m/s and 3 m/s^2^ to 13 m/s^2^, respectively. V-TENG has great advantages in collecting micro mechanical energy, and speed, acceleration detection. It has potential application prospects in the fields of automobile anti-theft, mechanical equipment operation monitoring and so on. But due to the poor recovery performance of the foam nickel-PDMS composite film after deformation, the repeatability of the detection signal is low, which can be improved by optimizing materials and structures. In addition, by shrinking the structure of V-TENG and arraying multiple V-TENGs, it is expected to improve the sensitivity, accuracy and linear range of V-TENG detection, which needs to be further studied in later work.

## 4 Experiment

### 4.1 Experimental materials

Polydimethylsiloxane and curing agent (Dow Corning Company, USA), foam nickel (thickness 0.2 mm Guangzhou Lithium Court Technology Co., Ltd.), nylon ball (diameter 25 mm Suzhou Yaster Seiko), and polypropylene test tube (Haimen Experimental Consumables Wholesale Center).

### 4.2 Fabrication of foam nickel-PDMS composite film

First, the 20 mL PDMS elastomer and curing agent are mixed in a mass ratio of 10:1 and stirred well with a glass rod. The PDMS mixed solution is then vacuum degassed for 20 min with a vacuum defoaming pump to remove the bubbles. Next, cut 3 cm * 3 cm of foam nickel, use ultrasonic instrument to wash and dry for 5 min, the PDMS mixed solution is evenly applied on it, to ensure that the PDMS mixed solution is fully coated with foam nickel, and pay attention to the surface thickness of not more than 0.5 mm, so that the surface of the PDMS film surface fully exposes the porous structure of foam nickel. Next, put the PDMS-coated foam nickel on the homogenizer table and set the speed to 1000 rpm and homogenize for 1min. After the homogenization is completed, put the sample into a vacuum drying box with a set temperature of 100 °C and a drying time of 30 min. Through the above steps, the production of foam nickel-PDMS composite film was completed.

### 4.3 Fabrication of V-TENG

The nylon ball with a diameter of 25 mm is placed into a polypropylene test tube, and the test tube mouth is covered with a 3 cm*3 cm foam nickel-PDMS composite film, and the wire is used to pass through the pores of the foam nickel, so that it is tightly connected and conducted, and finally the composite foam nickel-PDMS composite film and the wire are fixed with insulating tape to complete the fabrication of the V-TENG.

### 4.4 Characterization

V-TENG is driven by Linmot PO1-37X120-C/C1100 tubular linear motor, which uses Linmot-talk, the supporting drive control software of linear motor, to control the motion state of linear motor. Choose the Keithley DMM6500 digital multimeter to test open-circuit voltage, short-circuit current.

## Author contribution statement

Wang Peng: Conceived and designed the experiments; Wrote the paper.

Qianqiu Ni: Performed the experiments; Analyzed and interpreted the data.

Linfeng He: Performed the experiments.

Qingxi Liao: Contributed reagents, materials, analysis tools or data.

## Data availability statement

Data included in article/supplementary material/referenced in article.

## Additional information

No additional information is available for this paper.

## Declaration of competing interest

The authors declare that they have no known competing financial interests or personal relationships that could have appeared to influence the work reported in this paper.
